# Incidental findings on brain MRI among Chinese at the age of 55–65 years: the Taizhou Imaging Study

**DOI:** 10.1038/s41598-018-36893-0

**Published:** 2019-01-24

**Authors:** Shuyuan Li, Fang Fang, Mei Cui, Yanfeng Jiang, Yingzhe Wang, Xuhui Kong, Weizhong Tian, Min Fan, Ziyu Yuan, Jinhua Chen, Qi Yang, Fuzhong Xue, Jiucun Wang, Ming Lu, Xiaofeng Wang, Xingdong Chen, Li Jin, Weimin Ye

**Affiliations:** 1 International Peace Maternity and Child Health Hospital, Shanghai Jiao Tong University School of Medicine, Shanghai, China; 2 Fudan University Taizhou Institute of Health Sciences, Taizhou, China; 3 Department of Medical Epidemiology and Biostatistics, Karolinska Institutet, Stockholm, Sweden; 4 Institute of Neurology, Huashan Hospital, Fudan University, Shanghai, China; 5 State Key Laboratory of Genetic Engineering, Collaborative Innovation Center for Genetics and Development and MOE Key Laboratory of Contemporary Anthropology, School of Life Sciences, Fudan University, Shanghai, China; 6 Taizhou People’s Hospital, Taizhou, China; 7 Taixing Disease Control and Prevention Center, Taizhou, China; 8 Department of Epidemiology and Biostatistics, School of Public Health, Shandong University, Ji’nan, China; 9 Human Phenome Institute, Fudan University, Shanghai, China; 10 Clinical Epidemiology Unit, Qilu Hospital of Shandong University, Ji’nan, China

## Abstract

Asymptomatic brain abnormalities are common incidental findings on brain MRI in the elderly population and can be regarded as imaging markers of early stroke and dementia. We initiated the Taizhou Imaging Study (TIS) to examine the prevalence and correlates of incidental findings using brain MRI among an elderly population residing in a rural area of China. A total of 562 individuals, at the age of 55 to 65 years, participated in the TIS study with a response rate of 90%. The prevalence of lacunes, white matter hyperintensity (WMH), cerebral microbleeds (CMB), perivascular space, and intracranial arterial stenosis was 26.69%, 10.68%, 18.51%, 27.76%, and 12.81%, respectively. Age and hypertension were the major correlates of these incidental findings. Per each year increase in age, the risks of WMH and CMB increased by 15% and 14%. Compared to individuals with normal blood pressure, individuals with hypertension had an increased risk of all incidental findings, with the adjusted odds ratios of 2.28 to 5.45. Correlations of age, gender and body mass index with brain gray matter fraction were also observed. The high prevalence of these findings indicates a need of preventative strategy to help prevent future stroke and dementia in this population.

## Introduction

Ageing-related diseases, such as stroke and dementia, have posed great burden to our society. In China, in parallel to the dramatic population aging, stroke has become the leading cause of death and responsible for about one fifth of all deaths annually. There are at present about 7.5 million survivors of stroke in China and every year 2.5 million new stroke cases are diagnosed^1^. Similarly, the number of dementia cases increased from 3.68 to 9.19 million between 1990 and 2010 in China^2^. With the current lack of effective treatment strategies for stroke and dementia, it is essential to develop early detection techniques aiming to initiate preventive and therapeutic interventions in the early stage of these diseases.

It has been demonstrated that asymptomatic brain abnormalities, including structure and functional brain changes, are present years before the clinical onset of stroke and dementia and can indeed be visualized using brain magnetic resonance imaging (MRI)^3,4^. Along with the rapid improvement of MRI technologies, brain MRI has been included to an increasing degree in large population-based cohort studies, such as the Rotterdam Scan study^5^, the Framingham Heart study^6^, the Atherosclerosis Risk in Communities study^7^, the UK Biobank study^8^, and the German National Cohort study^9^. Previous studies have for example shown that lacunes, white matter hyperintensity (WMH), and cerebral microbleeds (CMB) are the most common incidental findings on brain MRI^5,10^. Although most of these findings are asymptomatic, numerous studies have demonstrated that they are associated with multiple mild clinical symptoms and signs, such as cognitive impairment, gait disturbance, and psychiatric disorders^11,12^, and are also related to future risks of stroke and dementia^13–15^. In 2011, the American Heart Association/American Stroke Association (AHA/ASA) published a statement about the vascular contributions to cognitive impairment and dementia, in which the association between MRI-based cerebrovascular disease-associated injury and vascular cognitive impairment was particularly emphasized^16^. In 2017, AHA/ASA published another statement demonstrating that silent brain infarcts and WMH are associated with the risk of future symptomatic stroke, and primary stroke prevention should be indicated in patients with silent brain infarcts, WMH, or CMB^17^. All the aforementioned facts indicate that MRI findings may be regarded as imaging markers of early stroke and dementia, emphasizing the significance of these image changes.

The prevalence and correlates of various incidental findings on brain MRI have been widely studied in the populations of North America and Europe^18–20^. However, the corresponding data are relatively scarce among general Chinese populations^17^. The aging society and the rapid economic growth during the past decades might have contributed to a rapid increase in the burden of ageing-related diseases in Chinese populations. Until now, only three population-based studies, including the Risk Index for Subclinical brain lesions in Hong Kong (RISK) study^21^, the Shanghai Aging Study (SAS)^22^, and the Shunyi study^23^, have made efforts to study incidental findings from brain MRI in the Chinese population^24^. Results of these studies showed that the prevalence of some incidental MRI findings was different from other populations^24^. However, all the three studies were conducted in the urban areas in China (Hong Kong, Shanghai, and Beijing), leaving the corresponding research questions in the rural areas unraveled.

Taizhou Longitudinal Study is a prospective, population-based study aiming at exploring the environmental and genetic risk factors of common chronic diseases in Chinese population^25^. Based on the Taizhou Longitudinal Study, we initiated the Taizhou Imaging Study (TIS) among an elderly population residing in a rural area of China. We targeted the study to a population at the age of 55–65 years, who had no clear clinical symptoms for stroke or dementia but might have a relatively high prevalence of preclinical abnormalities. In this paper, we aimed to report on the prevalence and correlates of incidental findings using brain MRI scan in this population.

## Methods

### Study design and study population

**Figure 1:**
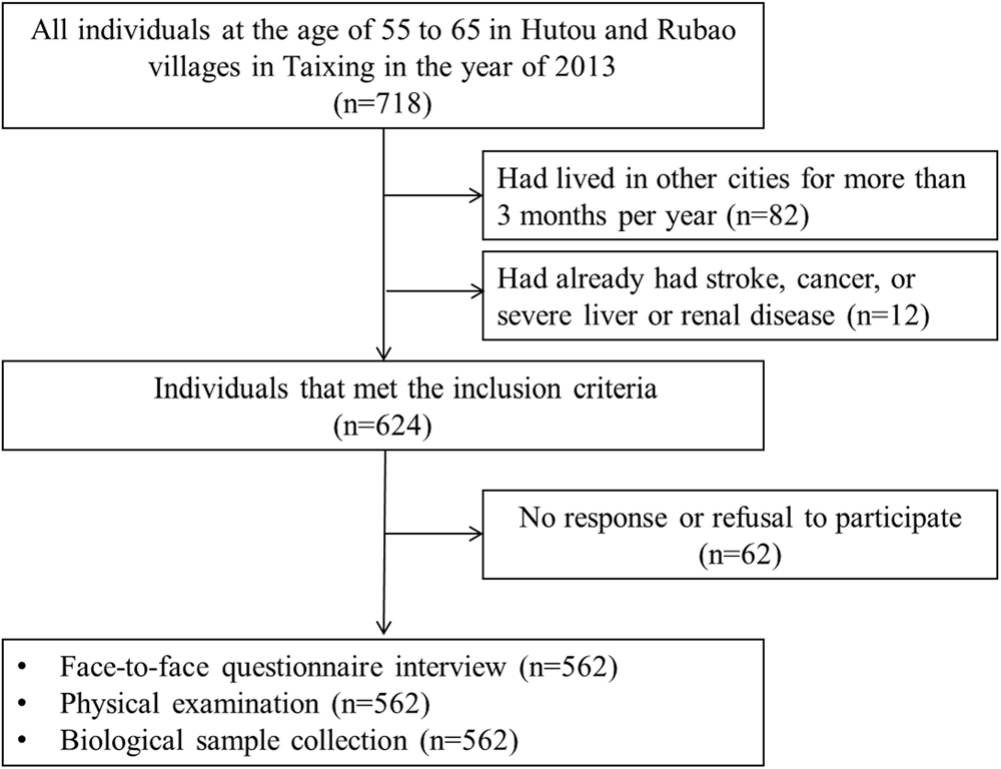
The enrolment procedures of the Taizhou Imaging Study (phase I).

The Taizhou Imaging Study (TIS) is an ongoing population-based, neuroimaging study initiated in March 2013 in Taizhou City, Jiangsu Province, China. The TIS study is nested within the Taizhou Longitudinal Study^25^. For the phase I of the TIS study, two villages with the highest participation rates in the Taizhou Longitudinal Study were selected from two towns of Taixing, a county-level city in Taizhou. The individuals in these two villages that fulfilled the following criteria were invited to participate in the study: (1) at the age of 55 to 65 years; (2) Han Chinese (self-reported, at least four generations) and had been a resident of these two villages for at least 10 years; 3) able to walk and communicate normally, and participate in the questionnaire survey and physical examination independently. Individuals that had had stroke, cancer, or severe liver or renal disease were considered not eligible for the study. Consequently, 624 individuals in the two villages were eligible for our study. Among them, 62 individuals did not respond to the invitation or refused to participate in the study. Overall, a total of 562 individuals participated and completed all procedures from March 2013 to January 2015, with a response rate of 90%. The responders and non-responders had no detectable differences in age and sex distribution. The enrolment procedures are shown in detail in Fig. 1.

### Questionnaire interview, physical examination and biological sample collection

After informed consent, a structured electronic questionnaire was administered by trained staff to obtain information on demographics and socioeconomic status, lifestyles, personal health status, family disease history, reproductive history (for women only), mental status, cognitive function (assessed using the Mini Mental Status Examination (MMSE)), and olfactory function (Table S1). All interviews were tape-recorded, and 5% of the recorded interviews were re-evaluated to ensure good quality of the interviews.

All participants received a full health check-up in Taizhou People’s Hospital, including anthropometric and blood pressure measurements, 12-Lead electrocardiography, chest X-ray, carotid artery ultrasound, bone mineral density, Ankle-to-brachial systolic blood pressure ratio and Pulse wave velocity, and brain MRI (Table S2).

Biological specimens including fasting blood (8-ml EDTA-anticoagulant and 8-ml non-anticoagulant), urine (15 ml), saliva (2~3 ml), and faeces were collected from each participant. The samples were processed using standard protocols. Briefly, EDTA-anticoagulant blood samples were subjected to centrifugation within one hour and aliquoted into ten barcoded cryogenic vials (four plasma samples, two buffy coat samples, and four red blood cell samples), while non-anticoagulant blood samples were aliquoted into six barcoded cryogenic vials (five serum samples, and one blood clot samples) after centrifugation. Saliva sample was mixed with 3 mL lysis buffer (50 mM Tris, pH 8.0, 50 mM EDTA, 50 mM sucrose, 100 mM NaCl, and 1% sodium dodecyl sulfate) and later transferred into one barcoded cryogenic vial. Urine and faeces were aliquoted into four and three barcoded cryogenic vials, respectively. All biospecimens were placed in coolboxes at 4 °C for less than 4 hours before being transported to the −80 °C freezers in the Biobank of the Fudan University Taizhou Institute of Health Sciences. One aliquot of serum was used for clinical testing of total cholesterol, triglycerides, low-density lipoprotein-cholesterol, high-density lipoprotein cholesterol, glucose, alanine amino transferase, total bilirubin, direct bilirubin, Uric acid, creatinine, urea nitrogen, and cystatin C by an automatic biochemical analyzer (TOSHIBA TBA-40FR) and all the other aliquots were stored at −80 °C freezers in the biobank.

### MRI Image acquisition and assessment

All MRI images were obtained from a 3-T Siemens Magnetom Verio syngo scanner, using an eight-channel head coil at Taizhou People’s Hospital. The same MRI protocol was performed on all participants, including mainly the following sequences: T_1_-weighted sequence, T_2_- and T_2_^*^-weighted gradient-recalled echo (GRE) sequence, fluid-attenuated inversion recovery (FLAIR) sequence, proton-density-weighted imaging (PDWI) sequence, perfusion weighted imaging (PWI) sequence, diffusion tensor imaging (DTI) sequence and time of flight (TOF) 3D angio sequence. The slice thickness was 1.5 mm for T_1_WI, 5.0 mm for T_2_*GRE, 0.6 mm for TOF-3D, 3.0 mm for T_2_WI, FLAIR, PDWI, DTI, and PWI, respectively (Table S3).

**Figure 2:**
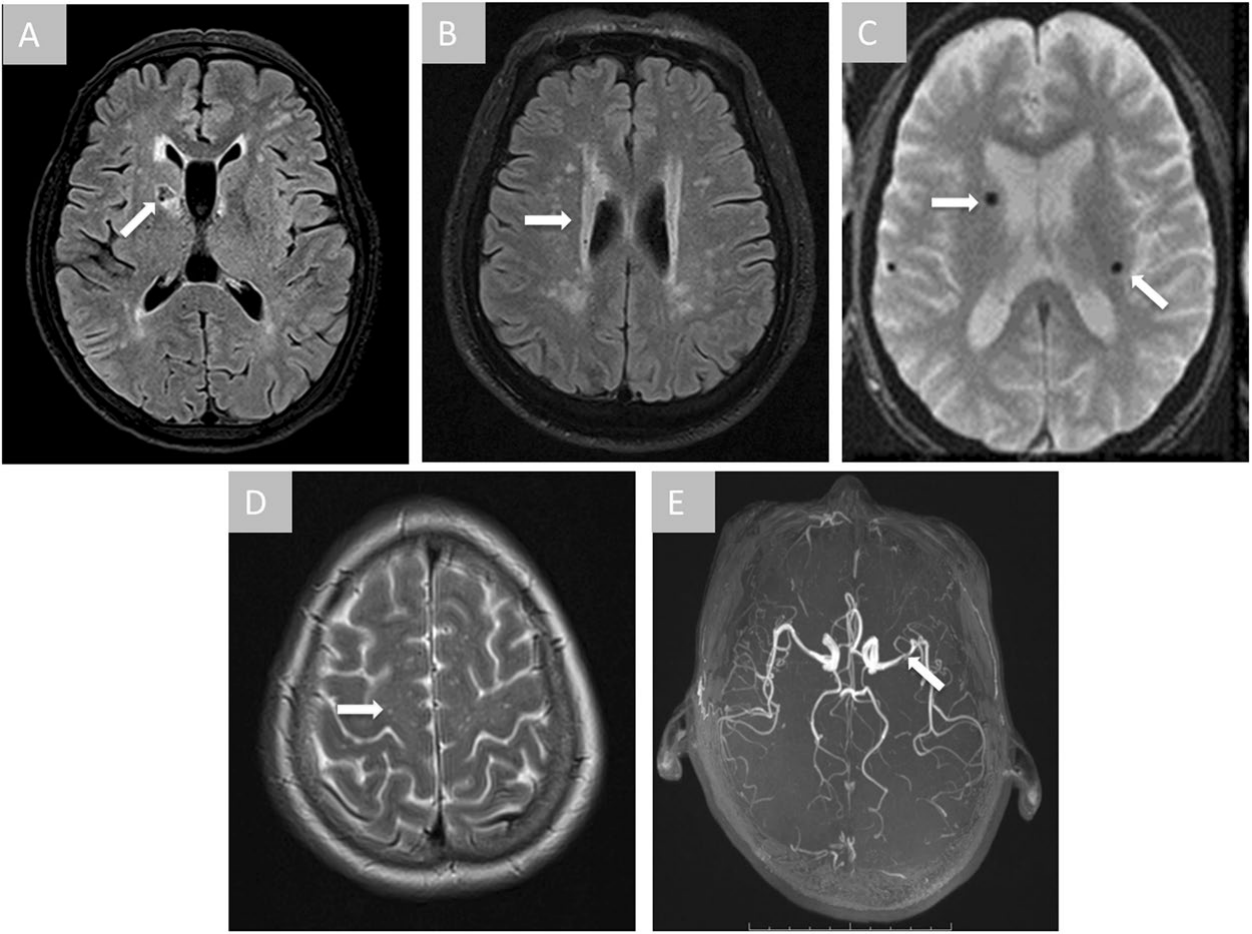
Incidental findings on brain MRI. Arrows indicate the abnormalities in each image. Panel A, lacunes; Panel B, white matter hyperintensity; Panel C, cerebral microbleeds; Panel D, perivascular space; Panel E, intracranial arterial stenosis.

All images were read by two experienced neurologists, Mei Cui and Qi Yang, who received small vessel diseases (SVD) imaging evaluation training strictly according to STRIVE criteria before the start of the study^26^. The neurologists were not aware of each other’s assessment and had no clinical information about the participants (disease history, carotid artery ultrasound, etc.), apart from age and sex of the participants and the date of MRI imaging. The neurologists read images independently through a standard digital picture archiving and communication system (PACS) and provided a good inter-rater reliability (kappa = 0.81). In case of controversy, a senior neuro-radiologist (Weijun Tang) was consulted for a final diagnosis. The primary diagnoses included lacunes, WMH, CMB, perivascular space (PVS), intracranial arterial stenosis (ICAS), and others (venous angioma, aneurysm, cavernous hemangioma, meningioma, empty sella, arachnoid cysts, ventricle cyst, 5th and 6th ventricle, 6th ventricle, widen septum pellucidum, and transparent compartment widened) (Fig. 2; Table S4). Lacunes, WMH, CMB, and PVS are the common markers of SVD and their diagnoses were made following the STRIVE criteria. As WMH is common in the elderly population, the discrimination of clinically significant lesions from the aging-related ones is important. We used therefore the Fazekas scale to separate WMH into grade 0–3^27^. Accordingly, WMH in grade 0–1 is regarded as a mild lesion that is more related to aging, while grade 2–3 suggests a severer abnormality that is more relevant to a pathological process (e.g., hypertension, cerebral amyloid angiopathy, etc.). In addition, previous studies have focused on small PVS with a maximum diameter of <2 mm on MRI to separate PVS from lacunes. However, recent studies lend strong support to the hypothesis that large PVS, which represents a more severe form of the lesion spectrum, serves as an independent MRI marker of SVD and may reflect processes related to cognitive impairment. Thus, we defined PVS as round or tubular deficits with CSF-like signal and a short axis of ≥3 mm in the subcortical area in this study. ICAS, as a common large vessel disease, was defined as local loss of signal from the internal carotid artery, middle cerebral artery, anterior cerebral artery, basilar artery, or vertebral artery on 3D-TOF MRA according to the WASID criteria^28^. All other diagnoses were made according to the criteria listed in the book of “Diagnostic Neuroradiology”^29^. The diagnoses were made based on MRI findings alone and were not confirmed by histology.

Moreover, the brain volume, including total intracranial volume, total gray matter volume, and total white matter volume, was estimated using FreeSurfer version 6.0.0 software package (http://surfer.nmr.mgh.harvard.edu/). The results of FreeSurfer were reviewed for accuracy by the consensus of two experienced neurologists (Yingzhe Wang and He Wang), and 31 of the 562 participants (5.52%) were excluded in the analyses of brain volume because of poor image quality, such as motion artifacts and inaccurate position. The brain parenchymal fraction (BPF) was defined as the ratio of brain tissue volume (total gray matter volume + total white matter volume) to total intracranial volume, and the brain gray matter fraction (BGMF) was defined as the ratio of total gray matter volume to total intracranial volume.

We delivered a MRI report to every participant. For participants with incidental findings that require additional clinical workup or medical treatment, such as aneurysm and meningioma, we provided them with a referral to a neurosurgeon or neurologist. Incidental findings that are believed not to be clinically relevant were not recorded as incidental findings, including simple sinus disease, pineal cysts, and ventricular asymmetry.

### Potential correlates of MRI markers

Current smokers were defined as individuals that reported regular smoking for at least six months, whereas former smokers were defined as individuals that reported to have smoked regularly for at least six months, but not during the year preceding the survey. Current smokers and former smokers were considered as ever smokers. Current alcohol drinkers were individuals consuming more than three drinks per week, while former alcohol drinkers were individuals that had drunk regularly for at least six months, but not during the year preceding the survey. Current alcohol drinkers and former alcohol drinkers were considered as ever alcohol drinkers. Body mass index (BMI) was calculated as body weight (kg) divided by squared height (m^2^). Hypertension was defined by systolic blood pressure ≥140 mm Hg or diastolic pressure ≥90 mm Hg, a previous diagnosis of hypertension, or use of antihypertensive drugs. Diabetes was determined by a fasting level of plasma glucose ≥7.0 mmol/L, a previous diagnosis of diabetes, or treatment with antidiabetic drugs. Hyperlipidemia was defined by plasma total cholesterol ≥5.2 mmol/L, triglycerides ≥1.7 mmol/L, a previous diagnosis of hyperlipidemia, or current use of lipid lowering drugs.

### Statistical analysis

Continuous variables were presented as mean ± standard deviation (SD) and were compared using Student *t* test or ANOVA. The variables with skewed distributions were described using median (interquartile range, IQR) and were compared using Kruskal-Wallis test. Categorical variables were summarized as number (percent) and were compared using Chi square test. Individuals with one of the specific MRI findings (lacunes, WMH, CMB, or PVS) were classified into the group with one SVD marker. Individuals with two, three, or four of these findings were grouped accordingly. The prevalence of each MRI finding and the SVD markers was calculated by age and sex. The associations of cardiovascular risk factors with MRI findings were assessed by binary logistic regression, using individuals without any MRI finding as the reference. The total score of MMSE was log-transformed to fit normal distribution. The associations of MRI findings with the log-transformed MMSE score were assessed using linear regression models. Fully adjusted models included age (per year), sex (male versus female), BMI (per kg/m^2^), education (per year), smoking (ever versus never), alcohol drinking (ever versus never), hypertension (yes versus no), diabetes (yes versus no), and hyperlipidemia (yes versus no). All analyses were performed using SAS statistical software (release 9.3, SAS Institute Inc, Cary, NC, USA) and *P* values < 0.05 were considered statistically significant.

### Ethical statement

This study was approved by the Human Ethics Committee of School of Life Sciences of Fudan University (number of Institutional Review Board approval: 469). This study was conducted in accordance with the approved protocol, and all participants provided written informed consent.

## Results

The basic characteristics of the studied population are summarized in Table 1. The mean age of the 562 participants was 59.25 ± 2.72 years, and 303 of the subjects (53.91%) were women. Males and females had comparable mean age, but females had higher BMI. Females were more likely to be less educated (median: 1 year), non-smokers (98.31%), and non-alcohol-drinkers (96.62%). The prevalence of hypertension, diabetes, and hyperlipidemia in the entire study population was 55.16%, 14.23%, and 54.80%, respectively. There was no clear difference in the prevalence of hypertension and diabetes by sex, although females had more hyperlipidemia. The median score of MMSE was 27, and was higher among males than females.

**Table 1:** Basic characteristics of the participants of the Taizhou Imaging Study.

	Total	Males	Females
N	562 (100)	259 (46.09)	303 (53.91)
Age, years, mean ± SD	59.25 ± 2.72	59.36 ± 2.74	59.15 ± 2.70
55–59, n (%)	301 (53.56)	133 (51.35)	168 (55.45)
60–65, n (%)	261 (46.44)	126 (48.65)	135 (44.55)
Education, years, median (IQR)	6 (0, 9)	9 (6, 9)	1 (0, 6)^*^
BMI, kg/m^2^, mean ± SD	24.09 ± 3.30	23.56 ± 3.02	24.54 ± 3.46^*^
Smoking status, n (%)			
Current smoker	195 (35.33)	191 (74.61)	4 (1.35)^*^
Former smoker	29 (5.25)	28 (10.94)	1 (0.34)
Non-smoker	328 (59.42)	37 (14.45)	291 (98.31)
Alcohol drinking status, n (%)			
Current drinker	162 (29.35)	153 (59.77)	9 (3.04)^*^
Former drinker	18 (3.26)	17 (6.64)	1 (0.34)
Non-drinker	372 (67.39)	86 (33.59)	286 (96.62)
Hypertension, n (%)	310 (55.16)	133 (51.35)	177 (58.42)
Diabetes, n (%)	80 (14.23)	33 (12.74)	47 (15.51)
Hyperlipidemia, n (%)	308 (54.80)	127 (49.03)	181 (59.74)^*^
MMSE, median (IQR)^#^	27 (23, 29)	29 (27, 30)	24 (20, 27)^*^

BMI, body mass index; MMSE, Mini-Mental Status Examination; IQR, interquartile range.

^#^Numbers of missing data were 19 for BMI, 10 for smoking status, 10 for alcohol drinking status, and 9 for MMSE.

^*^Differences between males and females were statistically significant (*P* < 0.05). The *P*-values were obtained using Student’s *t* test for continuous variables with normal or approximately normal distribution, using Kruskal-Wallis test for continuous variables with skewed distribution, and using chi-square test for categorical variables.

Lacunes and PVS were present in 150 (26.69%) and 156 (27.76%) of the participants (Table 2). No clear difference was found in the prevalence of these two MRI findings by age and sex. The prevalence of WMH-periventricular (Fezekas ≥2) and WMH-deep (Fezekas ≥2) was 10.68% and 8.72%, respectively, both of which were higher in the older age group. All individuals with WMH-deep had also WMH-periventricular. CMB was more prevalent in the older age group (23.37%) than younger age group (14.29%), and in females (21.78%) than males (14.67%). A higher prevalence of multiple SVD markers was observed in the older age group. The prevalence of ICAS was 12.81% and did not differ by age or sex. There was a decreasing trend of BGMF with increasing age, but there was no similar trend for BPF. The BGMF and BPF were greater in females than males. The prevalence of other MRI markers is shown in Table S4. None of the individuals with the reported venous angioma (n = 2), aneurysm (n = 1), cavernous hemanginoma (n = 1), empty sella (n = 11), and meningiomas (n = 5) reported symptoms.

**Table 2:** The distribution of common MRI markers among the participants of the Taizhou Imaging Study.

Findings	Entire study	Age, years			Gender		
		55–59	60–65	*P* ^*^	Males	Females	*P* ^*^
N	562	301	261		259	303	
Lacunes, n (%)	150 (26.69)	72 (23.92)	78 (29.89)	0.11	62 (23.94)	88 (29.04)	0.17
WMH, n (%)	60 (10.68)	22 (7.31)	38 (14.56)	**0.006**	26 (10.04)	34 (11.22)	0.65
Periventricular	60 (10.68)	22 (7.31)	38 (14.56)	**0.006**	26 (10.04)	34 (11.22)	0.65
Deep	49 (8.72)	16 (5.32)	33 (12.64)	**0.002**	19 (7.34)	30 (9.90)	0.28
CMB, n (%)	104 (18.51)	43 (14.29)	61 (23.37)	**0.006**	38 (14.67)	66 (21.78)	**0.03**
Brain stem	10 (1.78)	5 (1.66)	5 (1.92)	0.82	5 (1.93)	5 (1.65)	0.80
Basal ganglia	50 (8.90)	24 (7.97)	26 (9.96)	0.41	19 (7.34)	31 (10.23)	0.23
Cortical	69 (12.28)	27 (8.97)	42 (16.09)	**0.01**	25 (9.65)	44 (14.52)	0.08
PVS, n (%)	156 (27.76)	80 (26.58)	76 (29.12)	0.50	73 (28.19)	83 (27.39)	0.83
Number of SVD markers, n (%)							
0	298 (53.02)	169 (56.15)	129 (49.43)	**0.009**	143 (55.21)	155 (51.16)	0.19
1	149 (26.51)	86 (28.57)	63 (24.14)		69 (26.64)	80 (26.40)	
2	47 (8.36)	17 (5.65)	30 (11.49)		21 (8.11)	26 (8.58)	
3	45 (8.01)	19 (6.31)	26 (9.96)		16 (6.18)	29 (9.57)	
4	23 (4.09)	10 (3.32)	13 (4.98)		10 (3.86)	13 (4.29)	
ICAS, n (%)	72 (12.81)	43 (14.29)	29 (11.11)	0.26	28 (10.81)	44 (14.52)	0.19
ICA	5 (0.89)	4 (1.33)	1 (0.38)	0.23	4 (1.54)	1 (0.33)	0.13
MCA	45 (8.01)	27 (8.97)	18 (6.90)	0.37	15 (5.79)	30 (9.90)	0.07
ACA	5 (0.89)	1 (0.33)	4 (1.53)	0.13	1 (0.39)	4 (1.32)	0.24
BA	10 (1.78)	6 (1.99)	4 (1.53)	0.68	2 (0.77)	8 (2.64)	0.10
VA	18 (3.20)	8 (2.66)	10 (3.83)	0.43	8 (3.09)	10 (3.30)	0.89
BGMF, median (IQR), %^#^	37.8 (34.0, 40.2)	38.3 (34.9,40.5)	37.3 (33.4, 39.8)	**0.02**	35.7 (30.7, 37.7)	39.7 (37.7, 41.0)	**<0.0001**
BPF, median (IQR), %^#^	73.3 (71.1, 75.8)	73.5 (71.3, 75.6)	73.1 (70.8, 76.1)	0.26	72.8 (70.0, 76.5)	73.6 (71.7, 75.4)	**0.02**

WMH, white matter hyperintensity; CMB, cerebral microbleeds; PVS, perivascular space; SVD, small vessel diseases; ICAS, intracranial arterial stenosis; BGMF, brain gray matter fraction; BPF, brain parenchymal fraction; IQR, interquartile range.

^#^Numbers of missing data were 31.

^*^The *P*-values were obtained using Kruskal-Wallis test for continuous variables with skewed distribution, and using chi-square test for categorical variables.

Among all the correlates studied, age and hypertension are the major factors that were significantly related to the MRI findings (Table 3). Per each year increase in age, the risk of WMH and CMB increased by 15% (confidence intervals 2% to 31%) and 14% (confidence intervals 3% to 26%), respectively. Compared to individuals with normal blood pressure, those with hypertension had an increased risk of all SVD markers and ICAS, with adjusted ORs of 2.28 to 5.45. An increasing magnitude of the associations was observed with the increasing severity of SVD markers (ORs increased from 1.72 to 4.46). In addition, ever alcohol drinking was associated with ICAS, whereas age, sex, and BMI were associated with BGMF (Table 3).

**Table 3:** Associations of baseline characteristics with the MRI markers among the participants of the Taizhou Imaging Study.

MRI markers	Age (per year)	Gender (males vs females)	BMI (per kg/m^2^)	Education (per year)	Smoking (ever vs never smokers)	Alcohol drinking (ever vs never drinkers)	Hypertension (yes vs no)	Diabetes (yes vs no)	Hyperlipidemia (yes vs no)
	OR (95% CI)^#^								
Lacunes	1.06 (0.98, 1.14)	1.25 (0.46, 3.37)	0.99 (0.93, 1.07)	1.00 (0.94, 1.08)	0.70 (0.27, 1.85)	1.36 (0.70, 2.65)	**2.28 (1.43, 3.65)** ^**^	0.73 (0.38, 1.41)	0.94 (0.60, 1.47)
WMH	**1.15 (1.02, 1.31)** ^*^	0.76 (0.22, 2.55)	0.96 (0.88, 1.06)	1.01 (0.92, 1.12)	0.45 (0.13, 1.53)	1.14 (0.44, 3.02)	**5.45 (2.55,11.65)** ^**^	0.96 (0.41, 2.23)	1.01 (0.53, 1.94)
CMB	**1.14 (1.03,1.26)** ^*^	1.47 (0.49, 4.37)	0.97 (0.89,1.05)	1.02 (0.94, 1.10)	0.64 (0.22, 1.92)	1.21 (0.56, 2.63)	**2.98 (1.73, 5.13)** ^**^	0.55 (0.25, 1.22)	1.12 (0.67, 1.22)
PVS	1.03 (0.95, 1.12)	0.60 (0.24, 1.52)	0.97 (0.90, 1.04)	1.00 (0.94, 1.08)	0.42 (0.17, 1.06)	1.15 (0.59, 2.25)	**2.66 (1.68, 4.22)** ^**^	0.71 (0.37,1.38)	0.93 (0.59,1.45)
Number of SVD markers									
1–2 vs 0	1.03 (0.95, 1.10)	0.98 (0.45, 2.14)	1.00 (0.93, 1.06)	1.02 (0.96, 1.08)	0.71 (0.33, 1.51)	1.06 (0.60, 1.88)	**1.72 (1.15, 2.58)** ^*^	0.62 (0.35, 1.10)	0.91 (0.61, 1.34)
3–4 vs 0	**1.13 (1.00, 1,26)** ^*^	1.18 (0.38, 3.65)	0.97 (0.89, 1.06)	0.99 (0.90, 1.08)	0.88 (0.29, 2,68)	0.93 (0.38, 2.27)	**4.46 (2.25, 8.84)** ^**^	0.75 (0.34, 1.66)	0.82 (0.45, 1.49)
ICAS	0.95 (0.85,1.07)	1.00 (0.26,3.80)	1.03 (0.94, 1.12)	0.98 (0.90, 1.08)	0.29 (0.07, 1.14)	**3.25(1.04,10.2)** ^*^	**4.13 (2.11, 8.11)** ^**^	0.58 (0.24, 1.42)	1.51 (0.81, 2.83)
	β (SE) ^#^								
BGMF, %	**−0.17 (0.07)** ^*^	**3.75 (0.74)** ^**^	**0.15 (0.06)** ^*^	0.01 (0.06)	−0.09 (0.72)	−0.63 (0.55)	−0.61 (0.39)	−0.41 (0.53)	0.03 (0.38)
BPF, %	−0.02 (0.08)	0.39 (0.81)	0.05 (0.06)	0.01 (0.06)	0.31 (0.78)	−0.70 (0.60)	0.35 (0.42)	0.03 (0.58)	−0.22 (0.41)

WMH, white matter hyperintensity; CMB, cerebral microbleeds; PVS, perivascular space; SVD, small vessel diseases; ICAS, intracranial arterial stenosis; BGMF, brain gray matter fraction; BPF, brain parenchymal fraction.

^#^Covariates listed in the table were mutually adjusted.

^*^P < 0.05.

^**^P < 0.01.

No association was found between all the MRI findings and the MMSE score (Table 4).

**Table 4:** Associations of cognitive function assessed by MMSE with the common MRI findings among the participants of the Taizhou Imaging Study.

MRI markers	MMSE score (log-transformed)	
	β (SE)^#^	*P* ^#^
Lacunes	0.02 (0.02)	0.43
WMH	0.03 (0.03)	0.35
CMB	0.003 (0.02)	0.90
PVS	0.03 (0.02)	0.12
Number of SVD markers		
1–2 vs 0	0.02 (0.02)	0.28
3–4 vs 0	0.002 (0.03)	0.92
ICAS	0.007 (0.03)	0.80
BGMF, %	0.003 (0.002)	0.15
BPF, %	0.002 (0.002)	0.41

WMH, white matter hyperintensity; CMB, cerebral microbleeds; PVS, perivascular space; SVD, small vessel diseases; ICAS, intracranial arterial stenosis; BGMF, brain gray matter fraction; BPF, brain parenchymal fraction.

^#^Adjusted for age (per year), sex (male versus female), BMI (per kg/m^2^), education (per year), smoking (ever versus never), alcohol drinking (ever versus never), hypertension (yes versus no), diabetes (yes versus no), and hyperlipidemia (yes versus no).

## Discussion

Given the known clinical relevance of brain MRI incidental findings in the pathophysiology of stroke and dementia, understanding the burden of these preclinical lesions in the Chinese population will provide important information regarding the future burden of healthcare service in China. We found that several incidental findings of brain abnormalities, including lacunes, WMH, CMB, PVS and ICAS, were common in a rural population at the age of 55 to 65 years in China. Age and hypertension were further found as the major correlates of these incidental findings.

Lacunes, WMH, CMB, and PVS are the main MRI image markers of SVD, the most common vascular cause of dementia and contributes about one-fifth of all stroke cases worldwide^30^. Due to the different age and sex distributions of the study populations, and the different MRI acquisition and assessment procedures, comparison of the prevalence of these SVD markers in our study with others is challenging (please see a summary of previous findings in Table S5). Generally, the prevalence of lacunes appears to be higher in Asian countries^31^, and our results coincide with this. The prevalence of lacunes was 26.69% in our study, higher than that of Rotterdam Scan Study^20^ and Canadian Prospective Urban Rural Epidemiological (PURE)-MIND study^32^. In the Rotterdam Scan Study^20^, asymptomatic brain infarcts, which is another term of lacunes, was present in 7.2% of the study participants (n = 2000, mean age of 63.3 years). The prevalence of silent brain infarct in the age groups of 50–59 years (n = 324) and 60–69 years (n = 262) was 5.9% and 11.5% in the Canadian PURE-MIND study^32^. Corresponding studies in the healthy elderly population in China are scant. The prevalence of lacunes in the RISK study (n = 850, mean age of 71.4 years)^33^ and the SAS study (n = 321, mean age of 69 years)^24^ was 32.6% and 28.6%, similar to the results of the present study. The prevalence of WMH may vary with different race or ethnicity and increase with age^17,34^. Considering individuals at the same or similar ages, the prevalence of WMH-deep in the present study (8.72%) is similar to that of Austrian Stroke Prevention Study^34^ and PURE-MIND study (7%)^32^. However, the prevalence of WMH-periventricular was higher in our study (10.68%) compared to the abovementioned studies (2%)^17^. A high prevalence of WMH (36.8%) was also found in the SAS study, which was conducted in Shanghai, China^24^. This may partly be due to a higher prevalence of hypertension in our study (55.16%) and the SAS study (54.4%), compared to the Austrian Stroke Prevention Study (32%) and PURE-MIND study (24%). Microbleeds are more prevalent among elderly people, ranging from 6% among people at the age of 45–50 years to 36% among people at the age of 80 years and above^35^. The prevalence of CMBs was 18.51% in our study, which is similar to the reported prevalence of 16.8% among the participants at the age of 60–69 years in the Rotterdam Scan Study^35^. The prevalence of CMB was however lower in the Framingham Heart Study (8.8%; n = 1965, mean age of 66.5 years)^36^ and in Age Gene/Environment Susceptibility (AGES)-Reykjavik Study (11.1%; n = 1962, mean age of 76 years)^37^. Compared to lacunes, WMH, and CMB, few population-based studies have evaluated the prevalence of PVS, especially large PVS. The prevalence of 27.76% as noted in the present study is within the range of 16.2% as reported in the AGES-Reykjavik Study (n = 2612, mean age of 74.6 years)^38^ to 42% as reported in the Northern Manhattan Study (n = 1228, mean age of 71 years)^39^. Small PVS, defined as a maximum diameter of <2 mm in cross-section, was thought to be a normal phenomenon^40^ and can be visualized in the brain MRI of nearly all individuals of the HUNT-MRI study (100%; n = 997, 50–65 years)^41^. Previous studies have demonstrated that ICAS may account for about 10% of stroke cases in Northern America and European countries, whereas the corresponding estimate is 50% in China^42^. The prevalence of ICAS (12.81%) in the present study is similar to the prevalence reported in previous studies among Chinese in the Asymptomatic Polyvascular Abnormalities Community study (APAC) (13.2%; n = 5440, age ≥40 years)^43^, but higher than the prevalence reported among Caucasians in the Barcelona-Asymptomatic Intracranial Atherosclerosis (Barcelona-AsIA) study (8.6%; n = 933, mean age of 66.3 years)^44^. These two studies measured however ICAS through transcranial Doppler.

Several studies have found that age is a major risk factor for SVD markers and ICAS^17,45^. Although the participants of the present study were relatively young (55–65 years), we found clear correlations of age with WMH and CMB. With respect to cardiovascular risk factors, we found, in accordance with other studies, that hypertension was associated with increased prevalence of all the SVD markers and ICAS^33,45^. The main clinical manifestation of SVD is vascular cognitive impairment^46^. However, no statistically significant positive association was found between SVD and MMSE score in the present study. One possible explanation for the lack of association is the relatively young age of the present study population and the relatively high proportion of less educated individuals, especially among females.

To the best of our knowledge, this is the first population-based study estimating the prevalence and correlates of incidental findings on brain MRI in a rural Chinese population. The major strengths of our study include the high response rate, which guarantees a good representativeness of the findings, the use of high-resolution MRI, and the careful evaluation of the imaging markers. Our study has also some limitations. First, the evaluation of cognitive function using MMSE is not sensitive. Montreal Cognitive Assessment (MoCA) scale has therefore now been added to the phase II of the TIS study. Second, we only included people at the age of 55 to 65 years, whether or not these findings may apply to older people remains unknown. However, as we are currently expanding the TIS study and following all study participants longitudinally, we will be able to assess the prevalence of brain MRI incidental findings and their correlates above age 65, as well as to better assess the relationships of these incidental findings to cognitive impairment and dementia in the near future. Multiple follow-up procedures have now been established for the TIS study. First, the village doctors of the two villages are asked to document all health service use, hospitalization, and death of the participants in detail. These documents are reviewed by trained data collectors of the TIS study annually. Second, all participants are contacted every two years by phone or in person to provide information about new disorders occurring since the last contact. Finally, we also acquire mortality and medical insurance data for all the participants from the local Bureau of Public Security and Bureau of Social Security of Taizhou once a year. All data are collected and verified by trained data collectors of the TIS study. To obtain detailed information on the clinical characteristics of specific disorders, we will also review relevant medical records from the Taizhou People’s Hospital and Taixing People’s Hospital, two major hospitals in Taixing.

In conclusion, we revealed a high prevalence of MRI incidental findings in a rural Chinese population at the age of 55–65 years. The high prevalence of these findings urges for development of preventative strategy to reduce future burden of aging-related diseases, such as stroke and dementia, in Chinese elderly population.

## Supplementary information

Supplementary information

## Data Availability

The datasets generated and analyzed during the current study are available from the corresponding authors on reasonable request.
