# Supplementary material for: Incidental findings on brain MRI among Chinese at the age of 55–65 years: the Taizhou Imaging Study
Source: Sci Rep. 2019 Jan 24;9:464. doi: 10.1038/s41598-018-36893-0 (PMC6345793; doi:10.1038/s41598-018-36893-0)
Supplement: Supplementary file 1 — Supplementary information [file 41598_2018_36893_MOESM1_ESM.docx]

**Incidental findings on brain MRI among Chinese at the age of 55-65 years: the Taizhou Imaging Study**

Short title: Incidental findings on brain MRI in TIS

Shuyuan Li ^1,2*^, Fang Fang ^3*^, Mei Cui ^4^, Yanfeng Jiang ^2,5^, Yingzhe Wang ^4^, Xuhui Kong ^6^, Weizhong Tian ^6^, Min Fan ^7^, Ziyu Yuan ^2^, Jinhua Chen ^6^, Qi Yang ^4^, Fuzhong Xue ^2,8^, Jiucun Wang ^2,5,9^, Ming Lu ^10^, Xiaofeng Wang ^2,5,9^, Xingdong Chen ^2,5,9^, Li Jin ^2,5,9#^, Weimin Ye ^3#^

1. International Peace Maternity and Child Health Hospital, Shanghai Jiao Tong University School of Medicine, Shanghai, China;
2. Fudan University Taizhou Institute of Health Sciences, Taizhou, China;
3. Department of Medical Epidemiology and Biostatistics, Karolinska Institutet, Stockholm, Sweden;
4. Institute of Neurology, Huashan Hospital, Fudan University, Shanghai, China;
5. State Key Laboratory of Genetic Engineering, Collaborative Innovation Center for Genetics and Development and MOE Key Laboratory of Contemporary Anthropology, School of Life Sciences, Fudan University, Shanghai, China;
6. Taizhou People’s Hospital, Taizhou, China;
7. Taixing Disease Control and Prevention Center, Taizhou, China;
8. Department of Epidemiology and Biostatistics, School of Public Health, Shandong University, Ji'nan, China;
9. Human Phenome Institute, Fudan University, Shanghai, China.
10. Clinical Epidemiology Unit, Qilu Hospital of Shandong University, Ji'nan, China.

^*^ Joint first authors

^#^ Joint senior authors

Correspondence to:

Dr Xingdong Chen, School of Life Sciences, Fudan University, Songhu Road 2005, Shanghai, China. [**xingdongchen@fudan.edu.cn**](mailto:xingdongchen@fudan.edu.cn)

Prof Xiaofeng Wang, School of Life Sciences, Fudan University, Songhu Road 2005, Shanghai, China. **xiaofengwang71@163.com**

**Table S1.** Summary of questionnaire data collected at baseline in the Taizhou Imaging Study

| **Categories** | **Variables** |
| --- | --- |
| **Demographic and socioeconomic status** | Date of birth  Gender  Nationality  Marital status  Education  Occupational history  Individual and household incomes  Assets  Household composition  Living conditions: past and now  Health insurance coverage |
| **Lifestyles** | Smoking history, including passive smoking history  Exposure to indoor air pollution from cooking or heating fuel  Alcohol consumption  Tea consumption  Dietary history, including Food Frequency Questionnaire (FFQ), nutritional supplement, history of severe food shortage, and consumption of spicy food  Physical activity measured through International Physical Activity Questionnaire (IPAQ) |
| **Personal health status** | Disease history  Medication history  Surgery history  Bowel movement  Oral health  Height and weight: 10 years ago and at 20 years of age |
| **Family history** | Medical history of family members including spouse, parents, brothers, sisters, children, uncles, aunts, and grandparents |
| **Reproductive history**  **(for women only)** | Age of first menstrual period  Status of menstrual cycle  Menopause status  History of pregnancy  History of contraceptive use |
| **Mental status** | Self-rated mood status Traumatic life events Sleep quality Depression and anxiety  Stress |
| **Cognitive function** | Mini–Mental State Examination (MMSE) |
|  |  |
| **Olfactory function** | The Connecticut Chemosensory Clinical Research Center test (CCCRC) |

**Table S2.** Summary of clinical measurements collected at baseline in the Taizhou Imaging Study

| **Variables** | **Measurements** |
| --- | --- |
| **Anthropometry** |  |
| Height | GL-310, Shanghai huanxi apparatus factory, China |
| Weight | GL-310, Shanghai huanxi apparatus factory, China |
| Hip circumference | Standard tape measure |
| Waist circumference | Standard tape measure |
| Percentage of body fat | HBF-371, OMRON-Colin, Japan |
| **Blood pressure** | BM−091, Andon Health Co., Ltd., China |
| **12-Lead electrocardiography** | ECG-1350P, Nihon Kohden, Japan |
| **Chest X-ray** | DR VS/TH, Philips, the Netherlands |
| **Carotid artery ultrasound** | Acuson S2000, Simens, Germany |
| **Bone mineral density** | Lunar DPX NT-400157; GE Healthcare, USA |
| **Ankle-to-brachial systolic blood pressure ratio (ABI) and Pulse wave velocity (PWV)** | BP-203RPE Ⅲ, OMRON-Colin, Japan |
| **Brain magnetic resonance imaging (MRI)** | 3-T Magnetom Verio Tim scanner, Siemens, Germany |
| **Fasting blood biochemistry** | TBA-40FR, TOSHIBA, Japan |

| MRI sequence | Voxel size(mm) | FOV(mm) | TR(ms) | TE(ms) | Flip Angle(deg) | Slice Thickness(mm) |
| --- | --- | --- | --- | --- | --- | --- |
| T_1_WI | 0.9×0.9×1.5 | 220 | 21 | 7.65 | 30 | 1.5 |
| T_2_WI | 0.9×0.9×3.0 | 220 | 3220 | 93 | 150 | 3 |
| T_2_*GRE | 0.9×0.9×5.0 | 220 | 613 | 22 | 150 | 5 |
| FLAIR | 0.9×0.9×3.0 | 220 | 8000 | 94 | 150 | 3 |
| PDWI | 0.9×0.9×3.0 | 220 | 3220 | 17 | 150 | 3 |
| TOF-3D | 0.9×0.9×0.6 | 220 | 21 | 3.6 | 18 | 0.6 |
| DTI | 1.7×1.7×3.0 | 220 | 15000 | 83 | NA | 3 |
| PWI | 1.7×1.7×3.0 | 220 | 1240 | 50 | 90 | 3 |

**Table S3.** Scan parameters of the MRI sequences in the Taizhou Imaging Study

T_1_WI, T_1_ weighted imaging; T_2_WI, T_2_ weighted imaging; T_2_*GRE, T_2_ gradient-recalled echo; FLAIR, fluid attenuated inversion recovery; PDWI, proton-density weighted imaging; TOF, time of flight; DTI, diffusion tensor imaging; PWI, perfusion weighted imaging.

**Table S4**. The prevalence of other MRI findings among the participants of the Taizhou Imaging Study

| Findings | n (%) |
| --- | --- |
| Venous angioma | 2 (0.36) |
| Aneurysm | 1 (0.18) |
| Cavernous hemangioma | 1 (0.18) |
| Meningioma | 5 (0.90) |
| Empty sella | 11 (1.96) |
| Arachnoid cysts | 1 (0.18) |
| Ventricle cyst | 1 (0.18) |
| 5 and 6th ventricle | 1 (0.18) |
| 6th ventricle | 5 (0.90) |
| Widen septum pellucidum | 1 (0.18) |
| Transparent compartment widened | 2 (0.36) |

**Table S5.** Prevalence of brain MRI incidental findings reported in the present study and previous studies: a summary

| Incidental findings | Study | n | Mean age/ age group (years) | Prevalence (%) |
| --- | --- | --- | --- | --- |
| Lacunes | Present study | 562 | 55-65 | 26.69 |
|  | Rotterdam Scan Study ^1^ | 2000 | 63.3 | 7.2 |
|  | PURE-MIND study ^2^ | 324 | 50-59 | 5.9 |
|  |  | 262 | 60-69 | 11.5 |
|  | RISK study ^3^ | 850 | 71.4 | 32.6 |
|  | SAS study ^4^ | 321 | 69 | 28.6 |
|  |  |  |  |  |
| WMH | Present study | 562 | 55-65 | 10.68 |
|  | SAS study ^4^ | 321 | 69 | 36.8 |
| WMH-periventricular | Present study | 562 | 55-65 | 10.68 |
|  | Austrian Stroke Prevention Study ^5^ and PURE-MIND study ^2^ | 644 | 55-64 | 2 |
| WMH-deep | Present study | 562 | 55-65 | 8.72 |
|  | Austrian Stroke Prevention Study ^5^ and PURE-MIND study ^2^ | 644 | 55-64 | 7 |
|  |  |  |  |  |
| CMB | Present study | 562 | 55-65 | 18.51 |
|  | Rotterdam Scan Study ^6^ | 1350 | 60-69 | 16.8 |
|  | Framingham Heart Study ^7^ | 1965 | 66.5 | 8.8 |
|  | AGES-Reykjavik Study ^8^ | 1962 | 76 | 11.1 |
|  |  |  |  |  |
| PVS | Present study | 562 | 55-65 | 27.76 |
|  | AGES-Reykjavik Study ^9^ | 2612 | 74.6 | 16.2 |
|  | Northern Manhattan Study ^10^ | 1228 | 71 | 42 |
|  |  |  |  |  |
| ICAS | Present study | 562 | 55-65 | 12.81 |
|  | APAC ^11^ | 5440 | 55.2 | 13.2 |
|  | Barcelona-AsIA study ^12^ | 933 | 66.3 | 8.6 |

WMH, white matter hyperintensity; CMB, cerebral microbleeds; PVS, perivascular space; SVD, small vessel diseases; ICAS, intracranial arterial stenosis; PURE-MIND study, Canadian Prospective Urban Rural Epidemiological-MIND study; RISK study, Risk Index for Subclinical brain lesions in Hong Kong study; SAS study, Shanghai Aging Study; AGES-Reykjavik Study, Age Gene/Environment Susceptibility-Reykjavik Study; APAC, Asymptomatic Polyvascular Abnormalities Community study; Barcelona-AsIA study, Barcelona-Asymptomatic Intracranial Atherosclerosis study

**References:**

1 Vernooij, M. W. *et al.*, Incidental findings on brain MRI in the general population. *N Engl J Med* **357** 1821 (2007).

2 Smith, E. E. *et al.*, Early cerebral small vessel disease and brain volume, cognition, and gait. *ANN NEUROL* **77** 251 (2015).

3 Hilal, S. *et al.*, Prevalence, risk factors and consequences of cerebral small vessel diseases: data from three Asian countries. *J NEUROL NEUROSUR PS* **88** 669 (2017).

4 Mok, V. *et al.*, Race-ethnicity and cerebral small vessel disease - Comparison between Chinese and White populations. *INT J STROKE* **9A100** 36 (2014).

5 Schmidt, R. *et al.*, Heterogeneity in age-related white matter changes. *ACTA NEUROPATHOL* **122** 171 (2011).

6 Poels, M. M. F. *et al.*, Prevalence and Risk Factors of Cerebral Microbleeds An Update of the Rotterdam Scan Study. *STROKE* **411** S103 (2010).

7 Romero, J. R. *et al.*, Risk factors, stroke prevention treatments, and prevalence of cerebral microbleeds in the Framingham Heart Study. *STROKE* **45** 1492 (2014).

8 Sveinbjornsdottir, S. *et al.*, Cerebral microbleeds in the population based AGES-Reykjavik study: prevalence and location. *J Neurol Neurosurg Psychiatry* **79** 1002 (2008).

9 Ding, J. *et al.*, Large Perivascular Spaces Visible on Magnetic Resonance Imaging, Cerebral Small Vessel Disease Progression, and Risk of Dementia The Age, Gene/Environment Susceptibility-Reykjavik Study. *JAMA NEUROL* **74** 1105 (2017).

10 Gutierrez, J. *et al.*, Brain Perivascular Spaces as Biomarkers of Vascular Risk: Results from the Northern Manhattan Study. *AM J NEURORADIOL* **38** 862 (2017).

11 Zhang, S. *et al.*, Prevalence and risk factors of asymptomatic intracranial arterial stenosis in a community-based population of Chinese adults. *EUR J NEUROL* **20** 1479 (2013).

12 Lopez-Cancio, E. *et al.*, The Barcelona-Asymptomatic Intracranial Atherosclerosis (AsIA) study: prevalence and risk factors. *ATHEROSCLEROSIS* **221** 221 (2012).
